# Cognitive flexibility in a Tanganyikan bower-building cichlid, *Aulonocranus dewindti*

**DOI:** 10.1007/s10071-023-01830-w

**Published:** 2023-10-18

**Authors:** Maëlan Tomasek, Midori Stark, Valérie Dufour, Alex Jordan

**Affiliations:** 1 Cognitive and Social Ethology Team, UMR 7247, PRC, BAT 40, Campus CNRS, Physiologie de la Reproduction et des Comportements, INRAE, CNRS, IFCE, Université de Tours, 23 Rue de Loess, 67037 Strasbourg, France; 2 University of Konstanz, 78464 Constance, Germany; 3 Max Planck Institute of Animal Behaviour, 78467 Constance, Germany

**Keywords:** Teleost cognition, Decision rules, Behavioural inhibition, Preference, Wild animals

## Abstract

Cognitive flexibility, the ability to modify one’s decision rules to adapt to a new situation, has been extensively studied in many species. In fish, though, data on cognitive flexibility are scarce, especially in the wild. We studied a lekking species of cichlid fish in Lake Tanganyika, *Aulonocranus dewindti*. Males create sand bowers as spawning sites and maintain them by removing any objects falling into it. In the first part of our experiment, we investigated the existence of spontaneous decision rules for the maintenance of the bowers. We showed that if a snail shell and a stone are placed in their bower, fish prefer to remove the shell first. In the second phase of our experiment, we took advantage of this spontaneous decision rule to investigate whether this rule was flexible. We tested five individuals in a choice against preference task, in which the fish had to modify their preference rule and remove the stone first to be allowed to then remove the shell and have a clean bower. While there was no overall trend towards flexibility in this task, there was variation at an individual level. Some individuals increased their preference for removing the shell first, deciding quickly and with little exploration of the objects. Others were more successful at choosing against preference and showed behaviours suggesting self-regulatory inhibition abilities. Bower-building cichlids could therefore be a promising model to study cognitive flexibility, and other aspects of animal cognition in the wild.

**Supplementary Information:**

The online version contains supplementary material available at 10.1007/s10071-023-01830-w.

## Introduction

Cognitive flexibility has been defined as the ability to adapt cognitive processing strategies to face new and unexpected conditions in the environment (Cañas et al. 2000). It enables animals to modify their decision rules to adapt to new challenging conditions, which is crucial in rapidly changing environments or upon arrival to new environments (Snell-Rood 2013; Wright et al. 2010). Cognitive flexibility is also a marker for cognitive evaluation, along with numerosity, object permanence, or inferential skills (Burkart et al. 2017; Herrmann et al. 2007). Cognitive flexibility tests have been implemented to compare very distant taxonomic groups like pigeons, goldfish, and rats (Gonzalez et al. 1967; Mackintosh 1968; Rayburn-Reeves et al. 2013) to better understand the role (if any) of the physical, social, and ecological environment on the evolution of cognition in various animal species. However, in contrast to the large number of studies that test cognitive skills in endotherms, cognitive studies in ectotherms, including in fish, remain relatively scarce (Aellen et al. 2022). In this regard, a promising model system to ultimately study how adaptation to socio-ecological environment may shape cognitive flexibility is the Tanganyikan cichlid fish radiation, a system in which comparison can be made without strong phylogenetic distance bias. Lake Tanganyika, one of the African Great Lakes, is home to about 250 endemic species of cichlids with high variation in their life history and socio-ecological conditions (Ronco et al. 2021). Studies on fish cognition are, however, challenging. First, standard cognitive tasks that are often designed for other taxa need to be adapted so as to be meaningful for the fish (Kohda et al. 2019). Second, laboratory studies, while allowing for investigations in a controlled environment, may also lack ecological relevance compared to studies conducted in the wild (Boesch 2021; Janmaat 2019; MacDonald and Ritvo 2016; Salena et al. 2021). Complementarily to laboratory studies, scientists should therefore also aspire to a better understanding of the challenges naturally present in the environment of the different fish species and the way they cognitively resolve them.

Different types of tasks can be used to assess subject performances in cognitive flexibility. On one hand, some tasks test the ability to counteract a spontaneous preference. For example, the detour task requires animals to modify their path and to refrain from choosing the most direct trajectory to obtain a food reward placed behind a transparent partition (Kabadayi et al. 2018; Santacà et al. 2019; Sovrano et al. 2018; Triki and Bshary 2021). In cichlids, detour tasks have been successfully performed by Nile tilapias *Oreochromis niloticus* and some Tanganyikan species from the Lamprologini tribe (Brandão et al. 2019; Salena et al. 2022). Another type of task, the reverse reward contingency task, also requires individuals to override their spontaneous preference for the most desired reward. This task consists in offering the choice between a smaller and larger reward and then providing subjects with the option that they have not chosen. To solve the task, individuals thus need to learn to select the smaller option, against their spontaneous preference. Most species tested (e.g. chimpanzees, Boysen and Berntson 1995) fail in the standard version (but see sea lions, Genty and Roeder 2006), although repeated exposure or procedure modifications lead to improved performance in several species (Beran 2022; Beran et al. 2016; Boysen et al. 1999; Silberberg and Fujita 1996). Choosing against spontaneous preference has been reported in some fish species. Cleaner wrasse *Labroides dimidiatus* are able in the wild to refrain from picking on their client’s mucus which they prefer over their ectoparasites (Grutter and Bshary 2003). However, laboratory studies showed that they generally failed in a standard version of reverse reward contingency task (Danisman et al. 2010). Choosing against spontaneous preferences has also been documented in mate-choice copying where females can change their spontaneous preference towards a male after observing other females’ mate choices (Witte and Ryan 2002). On the other hand, some tasks like reversal learning tasks assess the flexibility of acquired preference rules. Cleaner wrasse succeed in such tasks (Aellen et al. 2022; Salwiczek et al. 2012; Triki and Bshary 2021) and in cichlids from the Lamprologini tribe most species succeed, albeit species differences in performances are not clearly understood (Bannier et al. 2017; Culbert et al. 2021; Fischer et al. 2021; Reyes-Contreras and Taborsky 2022; Salena et al. 2022).

To solve cognitive flexibility tasks, individuals need to demonstrate self-regulatory inhibition (also called behavioural inhibition; Beran 2018; Izquierdo et al. 2017), the ability to inhibit a normally favoured response (Beran 2018; Izquierdo et al. 2017). Some tasks can also be used to test the capacity to acquire concrete (building a simple association between a stimulus and the reward) versus abstract rules (connect the stimuli by an abstract relationship) (Rumbaugh and Pate 1984). Abstract rule learning can lead to highly flexible strategies such as win-stay/lose-shift responses (Rayburn-Reeves et al. 2013). More generally, these tasks are of interest because they make it possible to evaluate whether individuals have simply acquired knowledge about a given stimulus, or whether they have “learnt to learn” (Rumbaugh and Pate 1984; Shettleworth 2010).

**Fig. 1:**
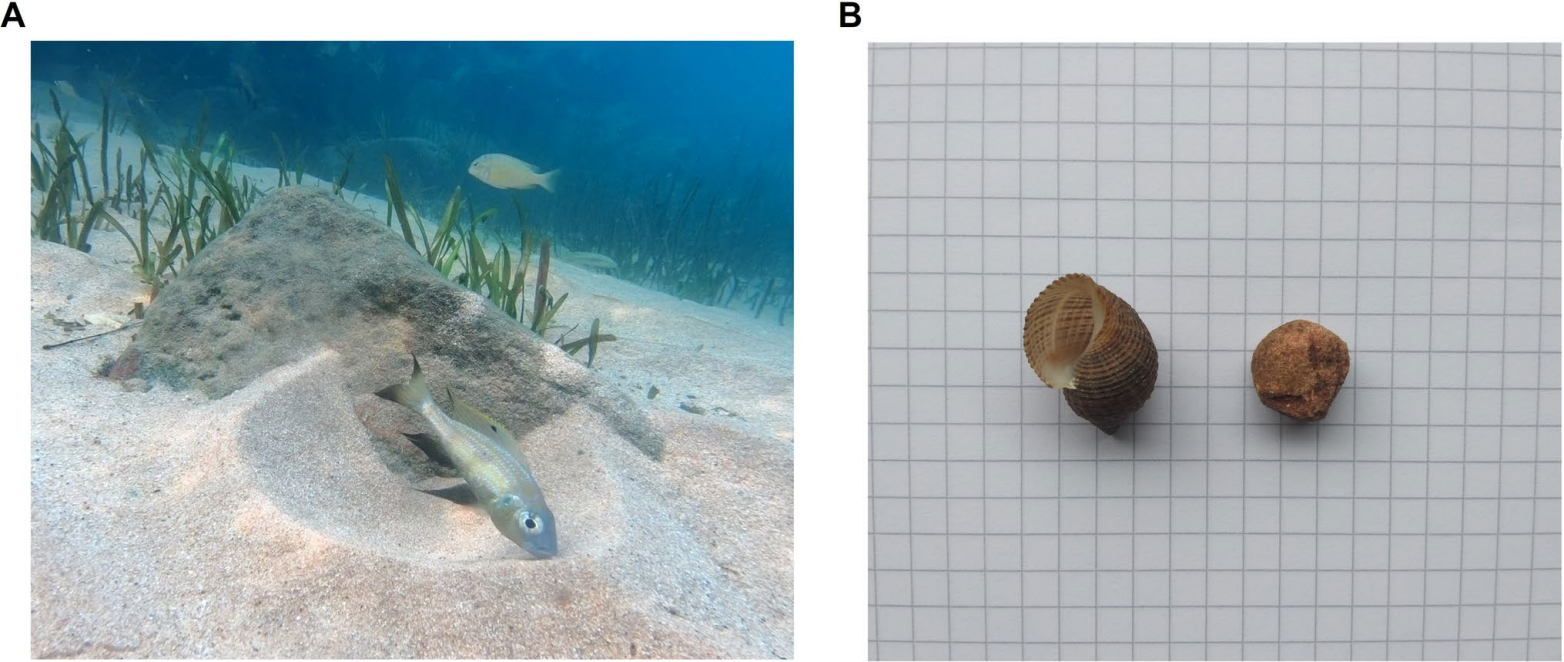
**A** Male *Aulonocranus dewindti* (Sweetboy) inside his bower. **B** *Lavigeria grandis* snail shell and stone used in our experiments (one square is 0.5 cm side)

While there are a handful of experiments that have assessed cognitive flexibility in wild populations of birds (Ashton et al. 2018; Bebus et al. 2016; Boogert et al. 2010; Cauchoix et al. 2017), almost all studies investigating fish cognitive flexibility have been conducted in laboratory settings with captive animals. In contrast, we here investigated the cognitive flexibility of a Tanganyikan cichlid species from the Ectodini tribe: *Aulonocranus dewindti* in completely natural wild conditions. Like many other cichlids, males build, maintain, and defend sand craters, called “bowers” that are used as spawning sites in a lekking system (Mckaye et al. 2001). Within these species, males construct bowers on or against a rock face, or in open sand (Fig. 1A), and after construction continue to maintain the bower by removing any foreign objects that lands in it (Konings 1998; Video 1). We took advantage of this spontaneous behaviour to design a new task to assess the cognitive flexibility of *A. dewindti*. In the first phase of our experiment, we looked for the existence of spontaneous decision rules in the maintenance of the bowers. We used two different objects that can be found in the environment (a snail shell and a stone) and presented them in the bower of wild males, investigating the existence of a preference in the order of removal of these objects. Decision rules for the maintenance of the bowers seem unlikely, as the behaviour of removing objects initially appears to be automatic, with fish immediately removing algae and waste as soon as they appear in their bower. A preference could, however, have adaptive value if the two objects were perceived differently hazardous by the animals. In most cases, it was clear this preference existed. We then evaluated in the second phase of our experiment whether they could modify this decision rule. To do so we discouraged the removal of the preferred object first by placing it back in the bower if removed, thus preventing the fish from completing the cleaning. If they removed the less preferred object first, they were then allowed to remove the last object and complete the cleaning of the bower. As for standard cognitive flexibility tasks, self-regulatory inhibition is needed to solve this task. Once again, due to the apparent automaticity of the behaviour, fish may lack the cognitive flexibility necessary for this task. However, given that other cichlids in captivity were able to solve reversal learning tasks or detour tasks, we sought to test the hypothesis that these cichlids may also demonstrate the ability to modify their decision rules through mechanisms like self-regulatory inhibition.

## Phase 1: preference task

### Material and methods

#### Subjects

Experiments were conducted on adult male *Aulonocranus dewindti* along the eastern Zambian shore of Lake Tanganyika (8°37′24.7′′ S, 31°12′02.9″ E) at a depth of 1–2 m and took place between 06:00 and 08:00 AM between 29th October and 13th November, 2022. Seven of twelve individuals tested were responsive during the first trials and were retained in this study. An individual qualified as responsive when it removed objects that were placed in the bower within five minutes. The responsive individuals typically removed items within two seconds. Individuals could be recognised from day to day because nesting males are highly philopatric and so were in the same location each day and could be further distinguished based on physical differences.

#### Experimental procedure

For each trial, an experimenter placed an empty *Lavigeria grandis* snail shell (1.71 g, 2 × 2  × 2cm) and a stone (1.86 g, 1.5 × 1.5 × 1cm) (Fig. 1B) in the bower of the targeted individual using two plastic poles to place the items from a distance (Supplementary video 1). The same shell and stone were retained throughout the experimental period. The shell and stone were placed simultaneously in the centre of the bower, with alternating positions so they were not always in the same orientation within the bower. When the experimenter approached, resident fish usually left the bower and therefore could not see the objects being placed inside the bower. The experimenter then moved away and the fish would return to their bower where they could remove both objects. An object was considered removed when it was placed beyond the rim of the bower; if a fish took an object which then slipped from its mouth and fell back in the bower, this object was not considered as removed. After the fish removed both objects, the experimenter waited between 20 and 40 s before starting a new preference trial (putting both objects inside the bower again). For each trial, the experimenter recorded which object the fish had removed first from the bower. The trials were video recorded with a GoPro7 at 25fps, either put on the ground at one or two metres from the bower or held by the experimenter at approximately one metre from the bower.

Two fish, Roger and Graham, were tested in a session of five trials in a row, for eight successive sessions (amounting to a total of 40 trials). Due to temporal and practical constraints, we could not conduct 40 trials for the other individuals (five fish). We checked whether they expressed the same preference by conducting two sessions (10 trials in total). The preference criterion for these fish to be included in the next phase was to remove the shell first in at least seven trials out of ten.

#### Statistics

All statistical analyses were done using R (version 4.2.2). A two-tailed exact binomial test was performed to test preference for Roger and Graham (null hypothesis set at 50%, “stats” package in R). To include the other individuals with fewer trials in the analysis, we checked for a consistent preference at the group level using a generalised linear mixed-effect model (Count of first removal ~ Type of object + (1|Subject)) with a Poisson family. We then conducted a model selection to compare with the null model based on AICc (function “dredge”, “MuMin” package in R).

### Results

**Fig. 2:**
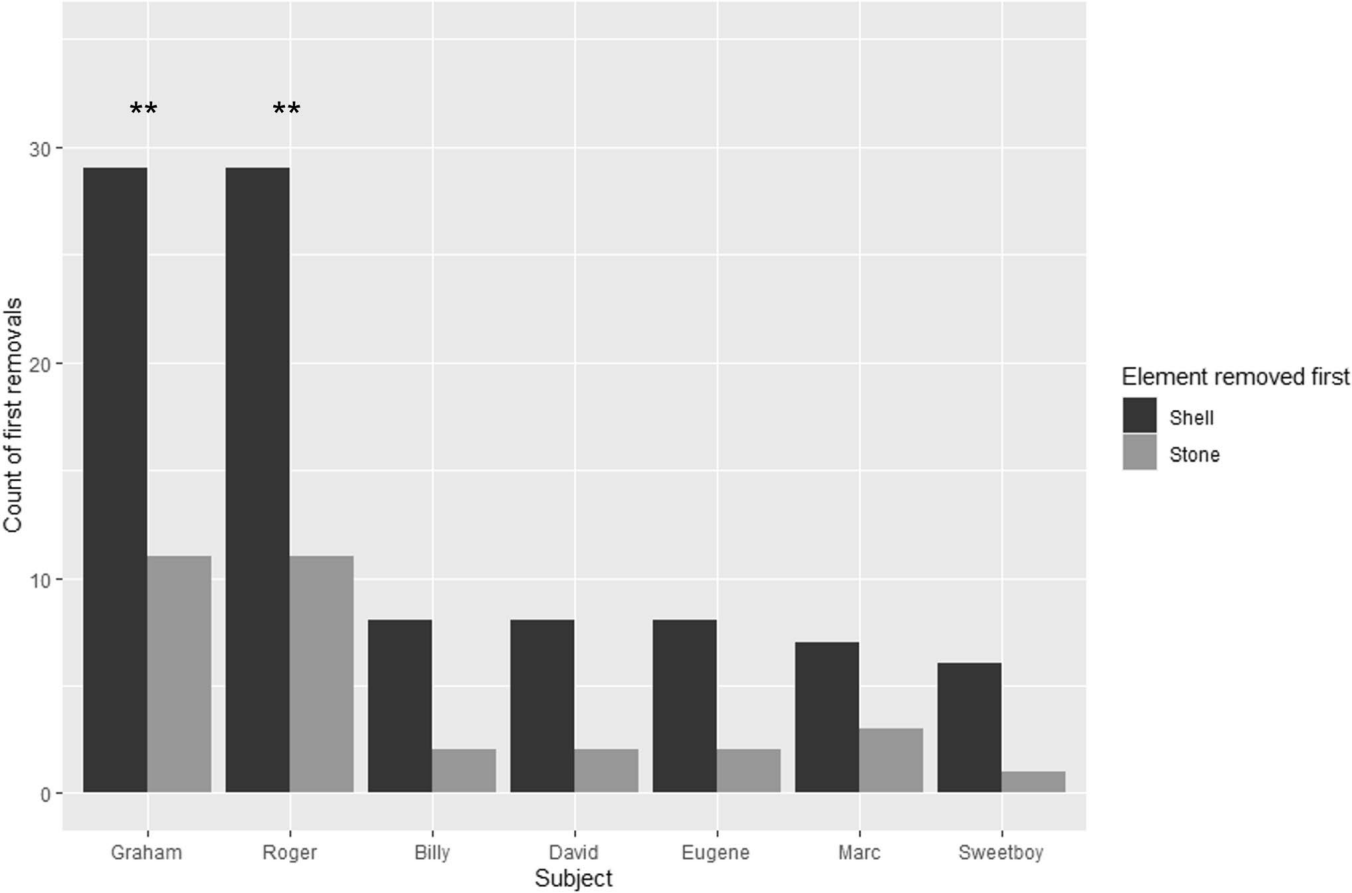
Preference of *A. dewindti* in the first object to remove when a snail shell and a stone are put in their bower. Statistical tests were conducted only on Graham and Roger (two-tailed exact binomial test, **p < 0.01). A generalised linear mixed-effect model (Count of first removals ~ Type of object + (1|Session)) with a Poisson family showed a significant bias in removing the shell first at the group level (see main text)

Both Graham and Roger showed a preference for removing the shell first (29 out of 40 tests for both, two-tailed exact binomial test, *p* < 0.01, estimated probability of removing the shell = 72.5%). This estimated probability was used further in our analyses of phase 2. Billy, David, and Eugene removed the shell first eight times out of ten, Marc seven times out of ten, and Sweetboy six times out of seven (Fig. 2). The median latency to take the first object out among all individuals was five seconds. We found a significant effect of the type of object in our model at the group level, showing a consistent bias to remove the shell first in all individuals (Supplementary table 1A, model with subject AICc = 86.0; null model AICc = 107.9). We thus confirmed a spontaneous decision rule in the maintenance of the bowers in *A. dewindti*, based on a reliable preference: individuals prefer removing the snail shell first, rather than the stone. We then investigated their cognitive flexibility by trying to modify this decision rule.

## Phase 2: choice against preference task

### Material and methods

#### Experimental procedure

To enter this phase, individuals had to show a preference for removing the shell first in phase 1. Two out of the seven individuals tested in phase 1 were dropped out in phase 2: Sweetboy because he became unresponsive in his second preference test session so did not complete ten trials, and Eugene because he left after the destruction of his bower by a storm in the beginning of phase 2.

The choice against preference task was conducted as follows: for each new trial, the experimenter placed the same shell and stone as in phase 1 in the bower of the fish. The removal of an object (shell or stone) by the fish ended the trial. If the fish removed the shell first, the experimenter quickly placed the shell back in the bower, which thus remained unclean (negative outcome), and a new trial began immediately. A “test” consisted of the succession of trials it took the fish to succeed in removing the stone first, up to a maximum of 20 trials per test. If, at a given trial within a test, the fish removed the stone first, he was then allowed to remove the shell as well, succeeding in cleaning the bower (positive outcome). This success also ended the test, and the fish was then given a minimum of three minutes where the experimenter left the area before coming back to start a new test.

**Fig. 3:**
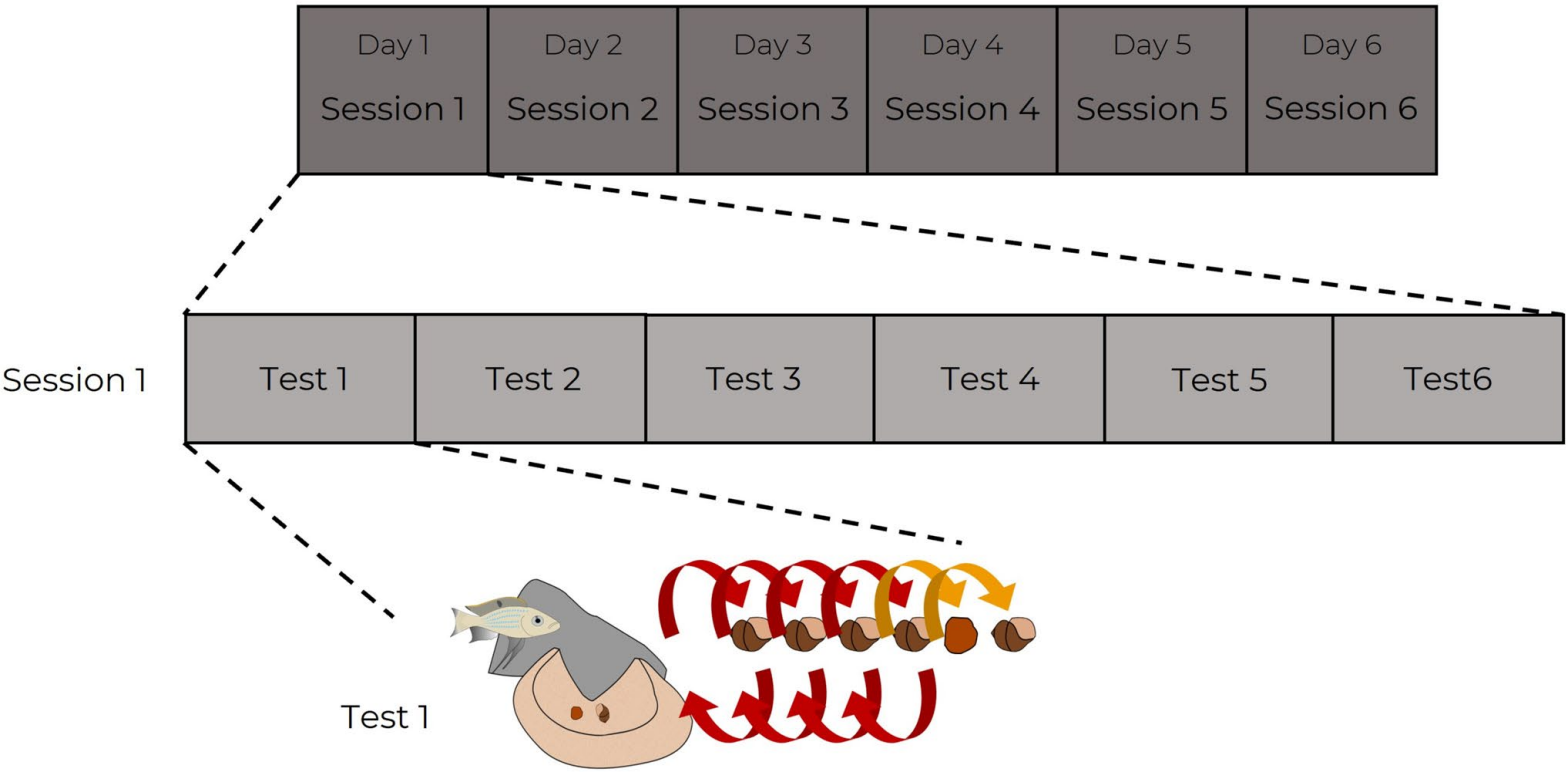
Experimental plan of phase 2. Six sessions of six tests were conducted in every individual. Each test consisted in a sequence of trials (a choice between the shell and the stone to remove) until the subject removed the stone. A maximum of 20 trials per test were conducted

If an individual had removed the shell first 20 consecutive times, without ever removing the stone first, then the experimenter took back both objects and ended the test. A “session” consisted of six tests done on the same day. Six sessions were conducted per individual, i.e. six consecutive days of six tests each (Fig. 3). Only complete sessions (of six tests) were kept in the analyses. In some sessions, fish became unresponsive and stopped engaging with the task. Two such sessions happened with David (one was dropped out after three tests, and the other one after five tests) and one with Marc (its first session was dropped out after three tests). We still managed to complete six sessions for David, but only five for Marc.

For each test, we scored from the videos the number of trials needed to remove the stone first, the actions oriented towards the objects inside or outside the bower (checking, touching, nudging, or other object-oriented actions, see supplementary material) according to the type of object, and the decision time. The decision time was defined as the time between the individual “seeing” the objects in the bower (either when the fish came back and crossed the rim of the bower or when the fish clearly switched direction to swim towards the bower, while being at a height enabling him to see inside) and the removal of an object (the object crossing the rim of the bower).

#### Statistics.

##### Overall performance

We investigated whether the performance in the task could be solely explained by the estimated preference displayed in the preference task (removing the shell 72.5% of the times and the stone 27.5% of the times). Using a geometric distribution, we calculated that if initial preferences explained the choice, it would be statistically unlikely (less than 5% chance) to observe tests requiring seven trials or more to remove the shell (see supplementary material). Requiring more than seven trials to remove the stone first would therefore be interpreted as that individual having significantly increased its preference for removing the shell first compared to phase 1. Below this criterion (fewer than seven trials to remove the stone first), it is not possible with a geometric law to know if choices are only guided by their initial preference or if the individuals have modified this preference. To explore a possible increase in the preference to remove the stone first, we instead focused on behavioural responses that could suggest such a process.

##### Inter-individual differences

We investigated inter-individual differences in the general success in the task (number of trials needed to remove the stone first). We ran a generalised linear mixed-effect model (Number of trials needed to remove the stone first ~ Subject + (1|Individual session)) with a Poisson family distribution. We then conducted a model selection to compare with the null model based on AICc (function “dredge”, “MuMin” package in R) before doing post hoc analyses [function “lsmeans”, “lsmeans” package in R (Lenth 2016)].

We then investigated inter-individual differences using two behavioural indices: the time taken to remove an object from the bower (either object) and the number of object-oriented actions inside the bower before making a choice. Decision time is a parameter that is often used in reversal learning tasks with longer decision times indicating a less automatic response (Olton and Samuelson 1974). We also interpreted the manipulations of the objects inside the bower as an exploration of the objects indicating a less automatic response as well. We therefore ran a generalised linear mixed-effect model (Decision time ~ Subject + (1|Individual session)) with a gamma family and a logarithmic link function and a generalised linear mixed-effect model (Number of manipulations ~ Subject + (1|Individual session)) with a Poisson family. For both models, model selection and post hoc analyses were conducted as described above.

Once the effects of the individuals were assessed for these parameters, we narrowed our analyses down to the individual level. We investigated behavioural differences in each individual between the situations where they removed the shell first (trial failure) or the stone first (trial success, choice against preference). We first investigated whether there were differences in the decision time between removing the shell and the stone. We ran generalised linear mixed-effect models with the “session number” as random factor (Decision time ~ Object removed + (1|Session)) per individual, with a gamma family and a logarithmic link function. We then investigated a possible difference in the number of actions oriented towards objects inside the bower whether the shell or the stone was taken out. We ran generalised linear mixed-effect models with the “session number” as random factor (Number of manipulations ~ Object removed + (1|Session)) per individual with a Poisson family.

##### Evolution of performance over time

To investigate a possible evolution of the performance at a large temporal scale (across sessions), we divided the experiment between “beginning” sessions (sessions 1–3) and “end” sessions (sessions 4–6). We then investigated whether there was a difference in success depending on the session epoch. We ran generalised linear models per individual (Number of trials needed to remove the stone first ~ Session epoch) with a Poisson family to investigate the number of trials needed to succeed. All models were subjected to model selection as described above. It is noted that a significant result can indicate (if it is in the direction of a decrease in the number of trials needed to remove the stone first) a learning process at a large temporal scale, whereas a non-significant result cannot be interpreted as an absence of learning.

To investigate a possible learning process at a shorter temporal scale (within a single session), we also divided tests between “beginning” tests (tests 1–3) and “end” tests (tests 4–6) and conducted the same analyses as described above but comparing between test epochs instead of sessions epochs. The same interpretations as the latter comparison can be made.

### Results

#### Overall performance

**Fig. 4:**
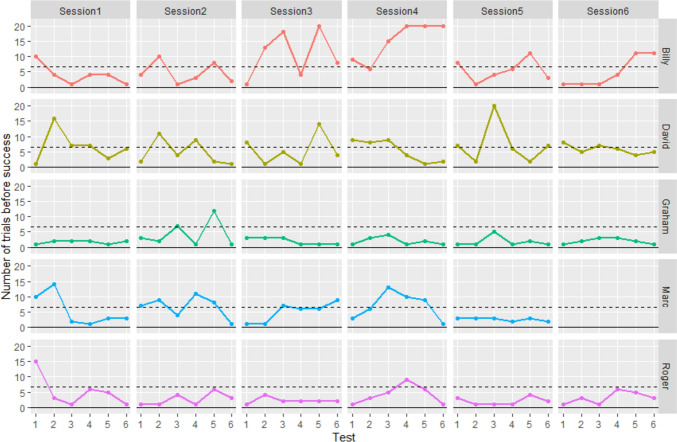
Overview of success (number of trials before removing the stone) in the different sessions. Tests above the dashed line had less than 5% of happening should the removal choices follow the preferences displayed in the preference task only (see Supplementary Material). Tests under the dashed line could be explained not only by this preference only but also by cognitive flexibility mechanisms (see supplementary materials). It is noted that tests reaching 20 trials mean that the fish removed the shell 20 times in a row and the test was stopped without the stone being ever removed

Three individuals performed multiple tests with seven trials or more: Billy in 44% of the tests (16 out of 36 tests), David in 42% (15 out of 36 tests), and Marc in 37% (11 out of 30 tests). In those tests, it was thus statistically unlikely that their preference for removing the shell first stayed equal to the preference displayed in phase 1, but instead that their preference for removing the shell first significantly increased. This was not observed in Graham and Roger (only 2 out of 36 tests where they took more than seven trials to remove the stone first; 1% of the tests; Fig. 4).

#### Inter-individual differences

**Fig. 5:**
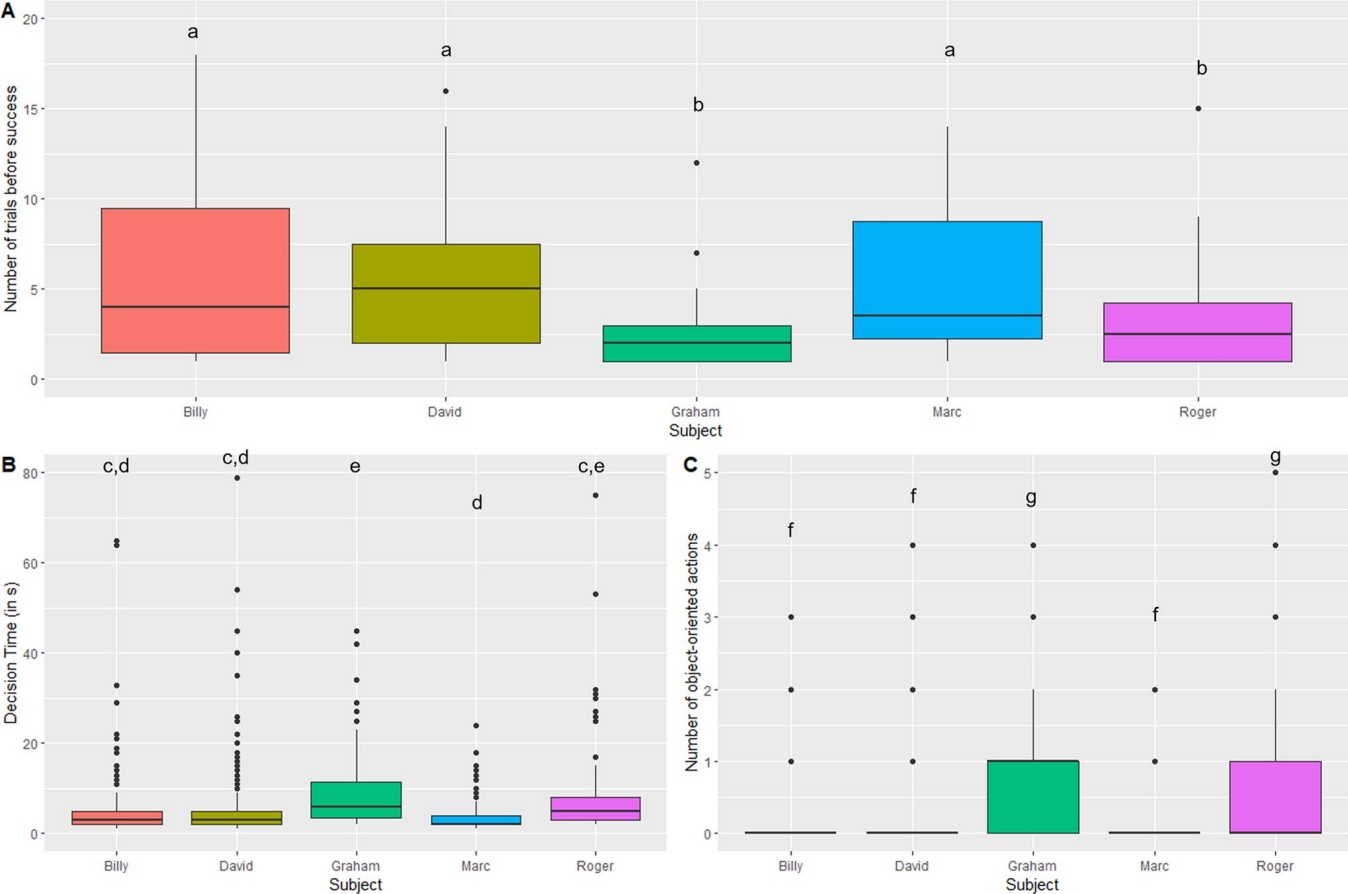
Inter-individual differences. **A** Differences between general successes in the experiment (number of trials needed to remove the stone first). **B** Differences in decision time. (It is noted that one trial for Graham at 217 s was not shown for a clearer graph). **C** Differences in the number of actions oriented towards the objects inside the bower before taking a decision (individuals with different letters present a significant difference, *p* < 0.05, post hoc least-square means differences from a generalised linear mixed-effect model (Variable of interest ~ Subject + (1|Individual session)), Poisson family for ranks of success and number of manipulations, gamma family with logarithmic link function for decision time)

**Fig. 6:**
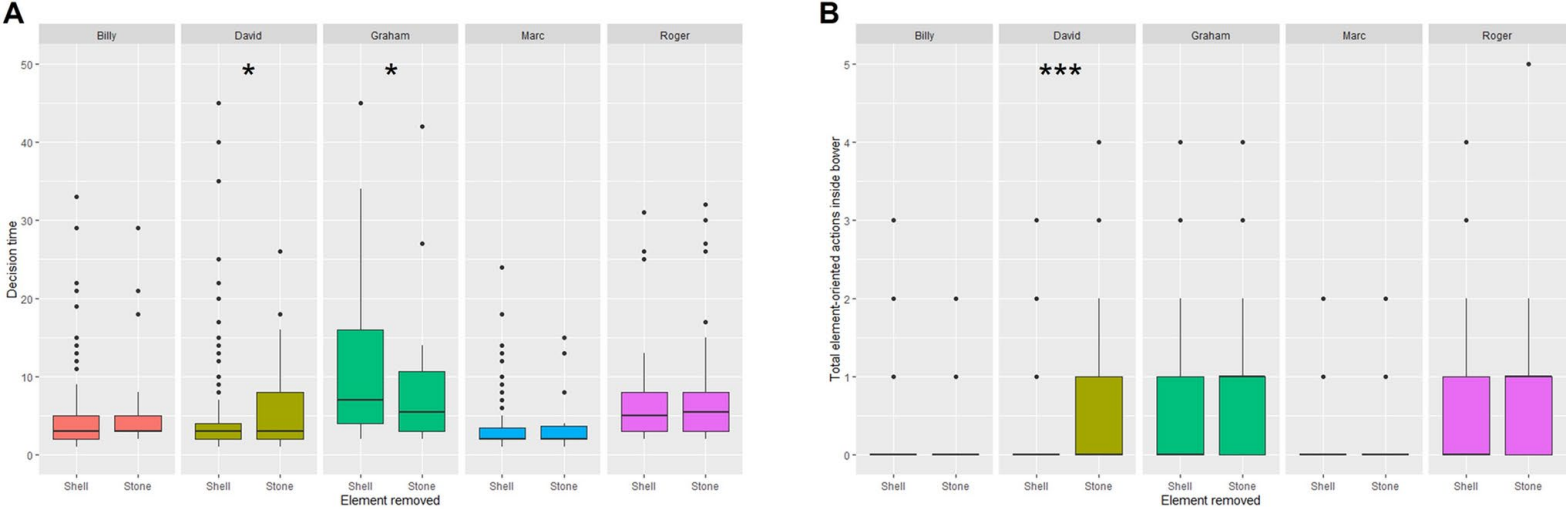
Exploration of behavioural differences during a success (removing the stone) or a failure (removing the shell). **A** Decision time depending on the object removed (generalised linear mixed-effect models per individual (Decision time ~ Object removed + (1|Session)), gamma family with logarithmic link, *p < 0.05). (It is noted that one trial for Graham at 217 s while he removed the shell was not shown for a clearer graph). **B** Actions oriented towards the objects inside the bower depending on the object removed (generalised linear mixed-effect models per individual (Number of manipulations ~ Object removed + (1|Session)), Poisson family, ***p < 0.001)

There was a significant effect of the individual on the general success in the task, i.e. the number of trials needed to remove the stone first (Supplementary table 2A, model with subject AICc = 902.7; null model AICc = 922.3). Post hoc analyses indicate that Graham and Roger succeeded in removing the stone first in significantly fewer trials than Billy, David, or Marc (Supplementary table 2A, Fig. 5A). There was also a significant effect of the individual on the decision time (Supplementary table 2B, model with subject AICc = 4367.6; null model AICc = 4380.1). Post hoc analyses indicate that Graham took longer to decide than all the others. Roger took longer than Marc but its decision time is not statistically different from David and Billy (Supplementary table 2B, Fig. 5B). Moreover, Graham displayed a significant difference between the decision time to remove the shell first and the stone first, taking a shorter time to remove the stone first than the shell first. On the contrary, David took significantly longer to remove the stone first than the shell first. All other individuals did not show any significant differences (Fig. 6A, Supplementary table 3A).

Finally, there was also a significant effect of the individual on the number of object-oriented actions inside the bower (Supplementary table 2C, model with subject AICc = 1127.6 null model AICc = 1161.3). Post hoc analyses indicate that Graham and Roger manipulated the objects more than Billy, David, and Marc (Supplementary table 2C, Fig. 5C). Only David displayed a significant difference between a number of manipulations of objects whether he removed the shell first or the stone first, doing more actions oriented towards the objects when he removed the stone first than when he removed the shell first. All other individuals did not show any significant differences (Fig. 6B, Supplementary table 3B).

#### Evolution of performance over time

**Fig. 7:**
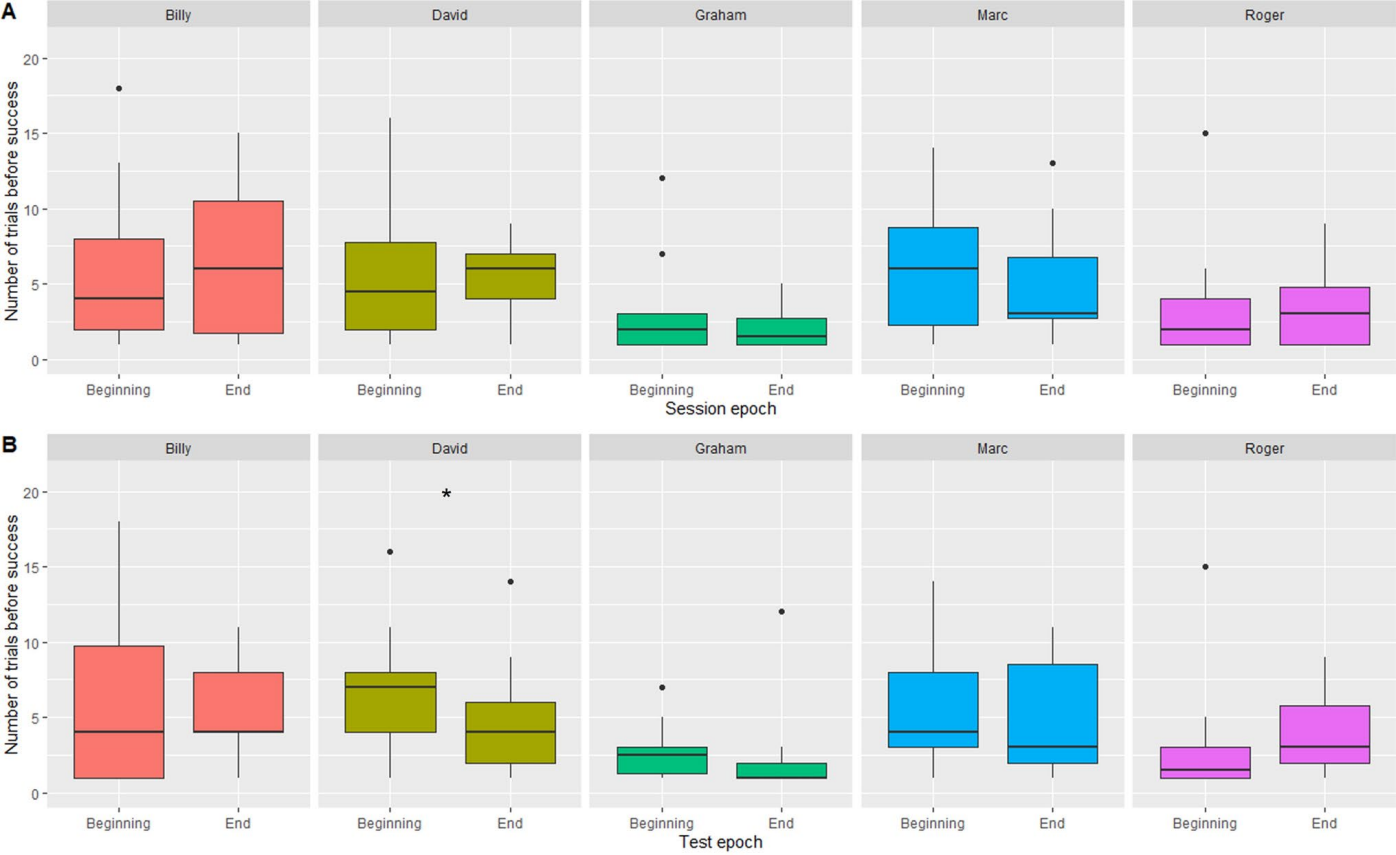
Exploration of the evolution of performance over time **A** at a large temporal scale (across sessions), evolution of the number of trials before removing the stone between sessions in the beginning of the experiment (sessions 1–3) and sessions in the end (sessions 4–6), **B** and at a shorter temporal scale (within each session, across tests), evolution of the number of trials before removing the stone between tests in the beginning of the sessions (tests 1–3) and tests in the end (tests 4–6) (generalised linear models (Number of trials before removing the stone first ~ Test epoch), Poisson family *p < 0.05)

For evolution at a large temporal scale (across sessions), no individual showed significant differences in the number of trials needed to remove the stone first between the “beginning” sessions and the “end” sessions (Supplementary table 4A, Fig. 7A).

For evolution at a short temporal scale (across tests, within a single session), only David took significantly more trials to remove the stone first in “beginning” tests than “end” tests (model with test epoch AICc = 199.3; null model AICc = 202.2, Supplementary table 4B, Fig. 7B).

#### Manipulations before decision

In some trials, an individual would manipulate the shell first (mostly touching or nudging it) and then decide to remove the stone (Supplementary Video 2). This behaviour is interesting because there is no apparent reason why a fish would have an object in or close to its mouth and not remove it and may therefore reflect behavioural inhibition. This behaviour could only be obtained in 36 opportunities maximum (or 30 maximum for Marc) because removing the stone leads to stopping the task, which happens six times per session. It was observed 13 times for Roger (36% of the opportunities), 9 times for Graham (25%), 7 times for David (19%), 4 times for Billy (11%), and twice for Marc (7%). As a comparison, we checked the trials where a fish would manipulate the stone and then decide to remove the shell. Roger did it 5 times (out of 80 opportunities, representing 6% of the opportunities), Graham 6 times (out of 47 opportunities, 13%), David 4 times (out of 230 opportunities, 2%), and Billy and Marc never did it (out of 233 and 165 opportunities, respectively).

## Discussion

Wild *Aulonocranus dewindti* fish are highly motivated to clean their bower and display a spontaneous decision rule for the maintenance of their bowers: they prefer to first remove a snail shell over a stone. We used this behaviour as an opportunity to conduct a choice against preference task to investigate the flexibility of such a decision rule. We found high inter-individual variability in the responses to the task: some individuals increased their preference to remove the shell first, while others showed some evidence of cognitive flexibility with respect to this otherwise rigid behavioural response. For these individuals, some behavioural indicators, including behaviours implying self-regulatory inhibition, suggest that they understood certain aspects of the task and were able to modify their preference rule.

In the first phase of our experiment, we examined whether spontaneous decision rules exist in bower maintenance behaviour. We found that *A. dewindti* displays a preference for removing an empty snail shell before a stone when we place both objects simultaneously in their bower. There are many snail species in Lake Tanganyika (Wilson et al. 2004), and many *Lavigeria grandis* snails can be found at our study site, moving on average at about 50 cm per day (Michel et al. 2007). Although we never observed a snail inside a bower, likely because they would be removed right away, we observed a snail moving along a bower rim once, suggesting snails can intentionally or inadvertently enter bowers. The reasons for such a preference rule remain unclear. Snails could be dangerous because they could eat sperm and eggs released in the bower, even though *L. grandis* are described as algae scrapers on rocks (Krings et al. 2021). Moving snails may also be more destructive of the bower than an inanimate object, hence their removal being done in priority to keep the bower as pristine as possible. The snail shell appeared to be more difficult to handle compared to the stone, with individuals frequently dropping the snail in an attempt to lift it, so the decision does not seem to rely on the shell being easier to carry than the stone. As the same two objects were used throughout the whole experiment, the decision could also be guided by their mere physical properties (size, weight, colour, etc.). Other cichlid species using shells as shelters show preferences for different shell attributes, which could also be salient for our study species and play a role in their preference (Bose et al. 2020). Further experiments using different shells and stones could shed light on the causes for this preference and tell for instance if fish are able to discriminate between a snail shell and a stone using categorisation abilities (Shettleworth 2010; in cichlids, Schluessel et al. 2012). This falls, however, out of the scope of this study in which finding one decision rule sufficed for cognitive flexibility tasks. Indeed, the preference for removing a shell before a stone in *A. dewindti* indicates that bower maintenance follows certain rules, making this species a good candidate to study cognitive flexibility by trying to modify those rules.

In the second phase of our experiment, we investigated whether the decision rule could be modified and the preference for removing the stone first increased by conducting a choice against preference task. We found strong inter-individual differences in the responses to the task: two individuals, Billy and Marc, often needed more than seven trials to remove the stone first. They failed to remove the stone first more often than the other individuals, interacted with the objects less than the other fish, and showed fast decision times for the first object they removed, suggesting that this was a more automatic behaviour than observed in other individuals. The preference for removing the shell first has in fact significantly increased in most trials compared to their initial preference displayed in the preference test (phase 1), as if repeatedly replacing the shell in the bower had focused their attention on this object.

Second, another individual (David) took fewer trials to remove the stone first in the last tests than in the first tests of his sessions, which indicates a possible learning process at the session scale (and hence short-term acquisition of the modification of the decision rule). Although like Billy and Marc he showed fast decision times and fewer manipulations of the objects, he interacted more with the objects and had longer decision times when he removed the stone first than when he removed the shell first. Also, several times, he nudged the shell before finally removing the stone first, a behaviour that might be an indicator of self-regulatory inhibition. This individual therefore appeared to understand more about the task than Billy and Marc and his performance improved over time within sessions.

Finally, two males, Roger and Graham, showed better overall performances compared to the other males. Their preferences for removing the shell first did not increase as they did in the other males, which may indicate a different choice mechanism compared to the other fish. From this statistical analysis only, it is not possible to conclude if their initial preference from phase 1 remained stable (removing the shell first approximately three times every four trials) or if it shifted towards a preference for the stone (which would mean that their decision rule has been modified; see supplementary material). However, several circumstantial lines of evidence suggest the decision rule was modified. First, they took fewer trials than the other fish to remove the stone first. Second, they took a longer time to decide before removing the first object. Third, they performed more actions oriented towards the objects inside the bower before taking a decision compared to the other fish. Finally, the observation that they touched the shell before finally removing the stone in a substantial number of tests is also consistent with a less automatic response and self-regulatory inhibition.

The preference rule in the order of removal of the objects therefore shows hints of being flexible, but not equally in all individuals. Some individuals did not seem to understand the task and increased their preference for removing the shell first, while others performed more successfully and showed signs of behavioural inhibition. These inter-individual differences could be explained by differences in experience. Roger and Graham had more trials in the first phase of our study and therefore more time to habituate to the interaction with the experimenter or to learn that the shell was empty and did not represent a threat. However, in total, the other three individuals had many more trials in the second phase and therefore more opportunities to interact with the empty shell. The difference in experience could also be due to the location of the bowers and the probability of encountering snails, which could be higher if the bower is against a rock face. However, Roger, Billy, and David, who showed lower capacity for flexibility, had bowers near rocks, while Graham and Marc’s bowers were in open sand. The differences between individuals could therefore rather be differences in cognitive flexibility abilities. It is common to observe individual variability in cognitive flexibility tasks (Bebus et al. 2016; Boogert et al. 2010) and more generally in animal cognition experiments (Boogert et al. 2018). Some studies have linked this variability in cognitive flexibility to evolutionary processes. For instance, poor performance in a reversal learning task can correlate with longevity and fitness benefits (Madden et al. 2018). Cognitive flexibility may also correlate with other factors such as personality (in zebra finches, Brust et al. 2013), cohesiveness (in cichlids, Culbert et al. 2021), or early exposure to predators (in cichlids, Bannier et al. 2017).

Contrary to other tasks, the preference rule that we investigate is not a strong one (there is not a choice that is preferred almost 100% of the time) which makes it difficult to interpret the results and to firmly conclude whether the decision rule has indeed been modified. While it is fairly easy to interpret that the preference for the shell has increased in case of poor performance, it is harder to separate whether the preference has remained stable or has been modified in case of better performances. We could only rely on behavioural indicators that reflect a modification of the rule and an understanding of the task. Those indicators, however, suggest that some individuals were indeed able to modify their decision rule. As we did not conduct transfer tasks, the mechanisms behind this modification remain unclear. The individuals could use concrete rules centred on the stimuli. Indeed, the shell could become aversive (and the stone thus more attractive) due to the repetitive replacement of the shell if removed and the action of the experimenter suddenly moving closer to the bower to put the shell back in, which might be very negatively perceived. However, there was no unusual delay in the removal of the shell once the stone was removed. Additionally, there was no general increase in the latency to remove the objects across tests or sessions. On the contrary, fish removed the objects quicker in the end sessions than in the beginning sessions (see supplementary materials). This suggests that the shell did not become aversive and that this explanation is unlikely. By default, both objects were connected by an abstract rule in our task (ordinality of the removals, rather than avoiding or preferring one of the two objects). Thus, the modification of the decision rule could have been guided by using a new abstract rule (now remove the stone first, instead of the shell first). Additional tests, involving transfer tests, may be a way to investigate this possibility further.

We conducted this study in the wild with untrained fish, who had never been exposed to cognitive training or testing before. While this allowed us to test cognition with the best possible subjects (i.e. subjects exposed to a large diversity of natural stimulations), it also had some pitfalls. It is worth keeping in mind that fish in our experiments were exposed to our task for a brief part of their day only (10–40 min per fish per day). Thus, our task may not have been salient enough compared to the diversity of events that occurred to them the rest of the time (including multiple removal of objects falling in their bower and being removed as usual). Further experiments should tackle issues raised in this study and improve the design of the task as well as the interpretability of the results. Our results are indeed encouraging to continue explore cognitive flexibility in *A. dewindti*: bower maintenance follows decision rules, and those rules might be flexibly modified but not equally so between individuals. This species presents several other advantages: they are the second most abundant species at our study site (Sturmbauer et al. 2008) and individuals stay at the same bower for several days in a row (more than 20 days in our study). Another cichlid species, *C. furcifer*, has also been shown to stay at the same bower for 42 to 46 days (Schaedelin and Taborsky 2006). As several different species of bower-building cichlids can be found at the same site, they could be a good avenue for conducting cross-species comparisons. Thus, despite the challenges associated with experiments in wild conditions, bower-building cichlids appear to be a promising model for cognitive studies in the wild, notably for comparative studies, and should help understand better the general cognitive skills in teleost fish.

### Supplementary Information

Below is the link to the electronic supplementary material.

Supplementary file1 (CSV 89 KB)

Supplementary file2 (CSV 0 KB)

Supplementary file3 (CSV 10 KB)

Supplementary file4 (CSV 7 KB)

Supplementary file5 (R 14 KB)

Supplementary file6 (MP4 124894 KB)

Supplementary file7 (MP4 245104 KB)

Supplementary file8 (DOCX 134 KB)

## Data Availability

Data and R script are available as supplementary materials.
