# Supplementary material for: Cognitive flexibility in a Tanganyikan bower-building cichlid, Aulonocranus dewindti
Source: Anim Cogn. 2023 Oct 18;26(6):1959–71. doi: 10.1007/s10071-023-01830-w (PMC10770232; doi:10.1007/s10071-023-01830-w)
Supplement: Supplementary file 8 — Supplementary file8 (DOCX 134 KB) [file 10071_2023_1830_MOESM8_ESM.docx]

**Supplementary materials**

**Methods: Number of trials needed to remove the stone first - statistics**

Two possible outcomes can occur in our choice against preference task: either the fish just keep on removing objects with the same initial preference they displayed in the preference task (removing the shell 72.5% of the times and removing the stone 27.5% of the times), or they can behave differently and show a change in this preference.

We investigate the first case, in which the removal of the objects only follows the preferences displayed in the preference task, which is our null hypothesis. Each trial can be considered as a Bernoulli trial where removing the shell is a failure and removing the stone is a success. We also hypothesize that each test is independent. We can then consider the variable X: “Rank of the success” in each test (i.e., the number of trials needed to remove the stone first in each test). This variable X follows a geometric distribution of parameter p = 0.275 (a geometric distribution is the probability distribution of the number X of Bernoulli trials needed to get one success; its parameter p is the probability of getting this success in each trial, here we take 0.275 because it is the value of the preference shown in the preference task). In such a distribution, the expected value for the number of independent trials to get the first success is $E\left( X \right)= \frac{1}{p}$. In our case, we have $E\left( X \right)\approx$ 3.6, which means that in each test, the fish should on average take three to four trials to remove the stone. We also calculated the number of trials $k$ so that $P\left( X>k \right)<0.05$. This means that there is less than 5% chance that a fish would take $k$ or more trials to remove the stone first, which gives here, $k \approx6.3$. Thus, if the fish removed objects only according to their initial preference, there would be less than 5% chance to obtain tests with seven trials or more.

Note that this statistical test is asymmetric as removing the stone is the endpoint of the experiment. Thus, only tests above seven trials can make us reject the null hypothesis and make us conclude that the preference for removing the shell first has increased. For tests below seven trials, we cannot reject the null hypothesis that the removal choices can be explained by the initial preferences only. In this case, we cannot firmly conclude whether the preference has remained stable compared to phase 1, or if it has shifted towards an increase of preference for the stone, and have to rely on behavioural hints that could indicate such a modification.

**Preference in removal of objects at the group level**

Due to time and practical constraints in the field, we could not obtain 40 trials in the preference tasks (phase 1) in all individuals. To check for a consistent preference at the group level, we conducted a generalised linear mixed-effect model.

| **Table 1A** glmer(Count of first removals ~ Type of object + (1\|Session), Poisson family) | | | | |
| --- | --- | --- | --- | --- |
| Model AICc = 86.0; null model AICc = 107.9 | | | | |
|  |  | Estimate ± SE | z-value | p-value |
| **Type of object** | Intercept | 2.41 ± 0.25 | 9.52 | **<0.001 ***** |
|  | Object removed first: Stone | -0.92 ± 0.19 | -4.82 | **<0.001 ***** |

| **Table 2A** Post-hoc analyses glmer(Number of trials before removing the stone first ~ Subject + (1\|Individual session), Poisson family) | | | | | |
| --- | --- | --- | --- | --- | --- |
| Model AICc = 902.7; null model AICc = 922.3 | | | | | |
| **Subjects** | **Billy** | **David** | **Graham** | **Marc** | **Roger** |
| **Billy** |  | 0.9774 | **<0.0001**  ******* | 0.9275 | **0.0016**  ******* |
| **David** |  |  | **<0.0001**  ******* | 0.9991 | **0.0112**  ***** |
| **Graham** |  |  |  | **0.0001**  ******* | 0.4023 |
| **Marc** |  |  |  |  | **0.0360**  ***** |
| **Roger** |  |  |  |  |  |

**Inter-individual differences analyses**

| **Table 2B** Post-hoc analyses glmer(Decision time ~ Subject + (1\|Individual session), gamma family with log link) | | | | | |
| --- | --- | --- | --- | --- | --- |
| Model AICc = 4367.6; null model AICc = 4380.1 | | | | | |
| **Subjects** | **Billy** | **David** | **Graham** | **Marc** | **Roger** |
| **Billy** |  | 1.0000 | **0.0239**  ***** | 0.8669 | 0.3150 |
| **David** |  |  | **0.0247**  ***** | 0.8646 | 0.3203 |
| **Graham** |  |  |  | **0.0017**  ****** | 0.8057 |
| **Marc** |  |  |  |  | **0.0478**  ***** |
| **Roger** |  |  |  |  |  |

| **Table 2C** Post-hoc analyses glmer(Number of manipulations ~ Subject + (1\|Individual session), Poisson family) | | | | | |
| --- | --- | --- | --- | --- | --- |
| Model AICc = 1127.6; null model AICc = 1161.3 | | | | | |
| **Subjects** | **Billy** | **David** | **Graham** | **Marc** | **Roger** |
| **Billy** |  | 0.7889 | **<0.0001**  ******* | 0.9997 | **<0.0001**  ******* |
| **David** |  |  | **<0.0001**  ******* | 0.9211 | **<0.0001**  ******* |
| **Graham** |  |  |  | **<0.0001**  ******* | 0.9673 |
| **Marc** |  |  |  |  | **<0.0001**  ******* |
| **Roger** |  |  |  |  |  |

**Supplementary table 2.** Inter-individual differences in **A.** success in the task (number of trials needed to remove the stone first), p-values of post-hoc analyses of least-square mean differences (function “lsmeans”, “lsmeans” package in R) of the indicated generalised linear mixed-effect model, **B.** decision time, p-values of post-hoc analyses of least-square mean differences of the indicated generalised linear mixed-effect model, and **C.** number of actions oriented toward the objects inside the bower (interpreted as explorative behaviours), p-values of post-hoc analyses of least-square mean differences of the indicated generalised linear mixed-effect model.

| **Table 3A** glmer(Decision time ~ Object removed + (1\|Session), gamma family with log link) | | | | |
| --- | --- | --- | --- | --- |
|  |  | Estimate ± SE | t-value | p-value |
| **Billy** | Intercept | 1.47 ± 0.10 | 14.06 | **<0.001 ***** |
|  | Object removed: Stone | 0.20 ± 0.14 | 1.435 | 0.151 |
| **David** | Intercept | 1.44 ± 0.22 | 6.77 | **<0.001 ***** |
|  | Object removed: Stone | 0.36 ± 0.14 | 2.57 | **0.0102 *** |
| **Graham** | Intercept | 2.61 ± 0.19 | 13.49 | **<0.001 ***** |
|  | Object removed: Stone | -0.48 ± 0.22 | -2.14 | **0.03 *** |
| **Marc** | Intercept | 1.26 ± 0.09 | 14.06 | **<0.001 ***** |
|  | Object removed: Stone | -0.06 ± 0.15 | -0.39 | 0.70 |
| **Roger** | Intercept | 2.01 ± 0.18 | 11.13 | **<0.001 ***** |
|  | Object removed: Stone | 0.08 ± 0.15 | 0.52 | 0.60 |

| **Table 3B** glmer(Number of manipulations ~ Object removed + (1\|Session), Poisson family) | | | | |
| --- | --- | --- | --- | --- |
|  |  | Estimate ± SE | z-value | p-value |
| **Billy** | Intercept | -1.95 ± 0.17 | -11.35 | **<0.001 ***** |
|  | Object removed: Stone | 0.12 ± 0.45 | 0.25 | 0.8 |
| **David** | Intercept | -2.28 ± 0.36 | -6.33 | **<0.001 ***** |
|  | Object removed: Stone | 1.47 ± 0.30 | 4.94 | **<0.001 ***** |
| **Graham** | Intercept | -0.19 ± 0.16 | -1.18 | 0.24 |
|  | Object removed: Stone | -0.04 ± 0.25 | -0.16 | 0.88 |
| **Marc** | Intercept | -1.93 ± 0.22 | -8.42 | **<0.001 ***** |
|  | Object removed: Stone | 0.32 ± 0.47 | 0.69 | 0.49 |
| **Roger** | Intercept | -0.43 ± 0.14 | -3.11 | **0.002 **** |
|  | Object removed: Stone | 0.21 ± 0.23 | 0.93 | 0.35 |

**Supplementary table 3.** Exploration of behavioural differences during a success (removing the stone) or a failure (removing the shell). **A.** Decision time depending on the object removed per individual, results of generalised linear mixed effect models. **B.** Number of actions oriented toward the objects in the bower depending on the object removed per individual, results of generalised linear mixed effect models.

**Learning processes**

| **Table 4A** glm(Number of trials before removing the stone first ~ Session epoch, Poisson family) | | | | |
| --- | --- | --- | --- | --- |
|  |  | Estimate ± SE | z-value | p-value |
| **Billy** | Intercept | 1.73 ± 0.10 | 16.96 | **<0.001 ***** |
|  | Session epoch: End | 0.12 ± 0.15 | 0.81 | 0.42 |
|  | Model AICc | 217.0 | | |
|  | Null model AICc | 215.3 | | |
| **David** | Intercept | 1.73 ± 0.10 | 17.52 | **<0.001 ***** |
|  | Session epoch: End | -0.05 ± 0.14 | -0.32 | 0.75 |
|  | Model AICc | 204.4 | | |
|  | Null model AICc | 202.2 | | |
| **Graham** | Intercept | 0.98 ± 0.14 | 6.80 | **<0.001 ***** |
|  | Session epoch: End | -0.32 ± 0.22 | -1.4 | 0.16 |
|  | Model AICc | 140.2 | | |
|  | Null model AICc | 140.0 | | |
| **Marc** | Intercept | 1.74 ± 0.10 | 17.70 | **<0.001 ***** |
|  | Session epoch: End | -0.17 ± 0.16 | -1.03 | 0.30 |
|  | Model AICc | 181.4 | | |
|  | Null model AICc | 181.2 | | |
| **Roger** | Intercept | 1.20 ± 0.13 | 9.33 | **<0.001 ***** |
|  | Session epoch: End | -0.07 ± 0.19 | -0.37 | 0.71 |
|  | Model AICc | 175.0 | | |
|  | Null model AICc | 172.9 | | |

| **Table 4B** glm(Rank of success ~ Test epoch, Poisson family) | | | | |
| --- | --- | --- | --- | --- |
|  |  | Estimate ± SE | z-value | p-value |
| **Billy** | Intercept | 1.79 ± 0.10 | 18.62 | **<0.001 ***** |
|  | Test epoch: End | -0.01 ± 0.15 | -0.09 | 0.93 |
|  | Model AICc | 217.6 | | |
|  | Null model AICc | 215.3 | | |
| **David** | Intercept | 1.87 ± 0.10 | 19.58 | **<0.001 ***** |
|  | Test epoch: End | -0.33 ± 0.15 | -2.26 | **0.02 *** |
|  | Model AICc | 199.3 | | |
|  | Null model AICc | 202.2 | | |
| **Graham** | Intercept | 0.96 ± 0.15 | 6.58 | **<0.001 ***** |
|  | Test epoch: End | -0.27 ± 0.22 | -1.20 | 0.23 |
|  | Model AICc | 140.8 | | |
|  | Null model AICc | 140.0 | | |
| **Marc** | Intercept | 1.75 ± 0.11 | 16.19 | **<0.001 ***** |
|  | Test epoch: End | -0.14 ± 0.16 | -0.87 | 0.39 |
|  | Model AICc | 182.8 | | |
|  | Null model AICc | 181.2 | | |
| **Roger** | Intercept | 1.04 ± 0.14 | 7.44 | **<0.001 ***** |
|  | Test epoch: End | -0.24 ± 0.19 | 1.30 | 0.20 |
|  | Model AICc | 173.5 | | |
|  | Null model AICc | 172.9 | | |

**Supplementary table 4.** Exploration of learning processes. **A.** Learning at a large temporal scale (across sessions): success in the task depending on beginning sessions (Sessions 1 to 3) or end sessions (Sessions 4 to 6), generalised linear models per individual as indicated. **B.** Learning at a short temporal scale (across tests): success in the task depending on beginning tests (Tests 1 to 3) or end tests (Tests 4 to 6), generalised linear models per individual as indicated**.**

**Actions oriented toward the objects**

Fish performed several actions oriented toward the objects, either inside their bower before making a choice, or outside their bower once the objects were removed. They could either check them out (intently swimming toward them but without touching them), touch them, nudge them, push them, or spit sand or waste on them. It was common for individuals to continue interacting with (e.g., spit sand on) the objects once the task was over and the objects outside of the bower (**Supplementary Video 2**).

| **Action** | **Location**  **(inside/outside bower)** | **Description** |
| --- | --- | --- |
| Check | Inside or outside | Obviously look at an object, swims towards it, directs his snout towards it, but does not make contact with the object |
| Touch | Inside or outside | Lightly make contact with an object with his snout |
| Nudge | Inside or outside | Takes an object between his lips and lightly pushes it, the object is moving whereas it does not move if the fish “touches” it (**Supplementary video 1**) |
| Push | Outside | Pushes an object that was put in a bower, the object rolls down the slope of the bower |
| Spit | Outside | Takes sand and/or algae waste in his mouth and spits it on an object that was removed from the bower |

**Supplementary table 5.** Ethogram of actions oriented toward the objects

We interpreted these behaviours as explorative behaviours, the individuals interacting with the objects without removing them or making a choice. Investigating these behaviours could reflect their comprehension of the task.

To investigate whether these actions were directed more toward one object than another, we ran a generalised linear mixed effect model (Number of actions ~ Object removed + (1|Subject)) with a Poisson family.


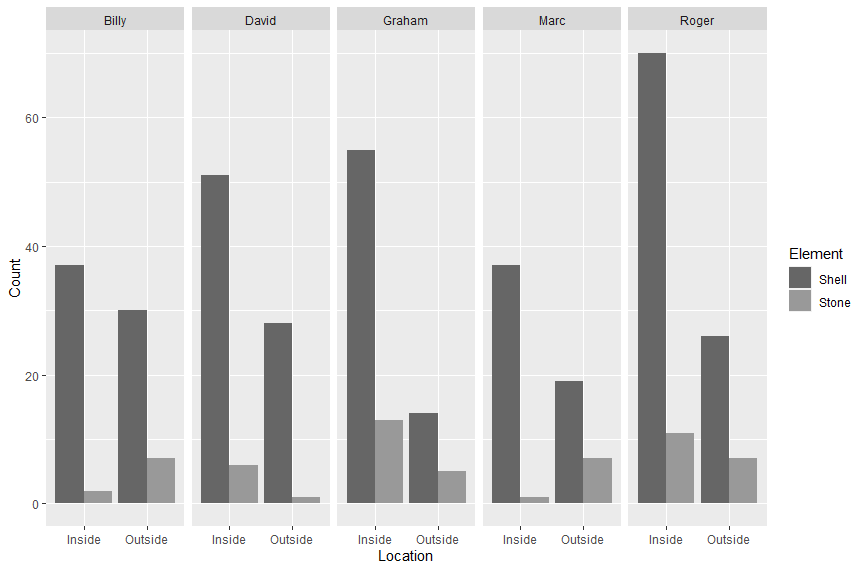
**Supplementary figure 1.** Number of actions oriented toward the objects depending on the object and the location in which the actions occur.

|  |  | Estimate ± SE | z-value | p-value |
| --- | --- | --- | --- | --- |
| **Result of the model** | Intercept | 3.59 ± 0.09 | 40.82 | **<0.001 ***** |
|  | Object removed: Stone | -1.81 ± 0.14 | -13.02 | **<0.001 ***** |
|  | Model AICc | 188.7 | | |
|  | Null model AICc | 431.2 | | |

**Supplementary table 6.** Results of a generalised linear mixed-effect model (Number of manipulations ~ Object removed + (1|Subject), Poisson family)

The fish significantly directed their actions more toward the shell than toward the stone. The shell is therefore very salient to the fish: they prefer to remove it first and they manipulate it more, whether inside or outside their bower. This suggests that it might be very hard for the fish to override their impulse to remove the shell first.

**Latency to remove objects across tests and sessions**

To investigate whether the negative outcome of putting the shell back if removed, and the experimenter moving closer to the bower which makes the fish swim away for it could lead to a strong aversion for the shell, and biases in our interpretations, we checked whether there was an increase in the latency to remove objects across tests and sessions.

*Across tests*


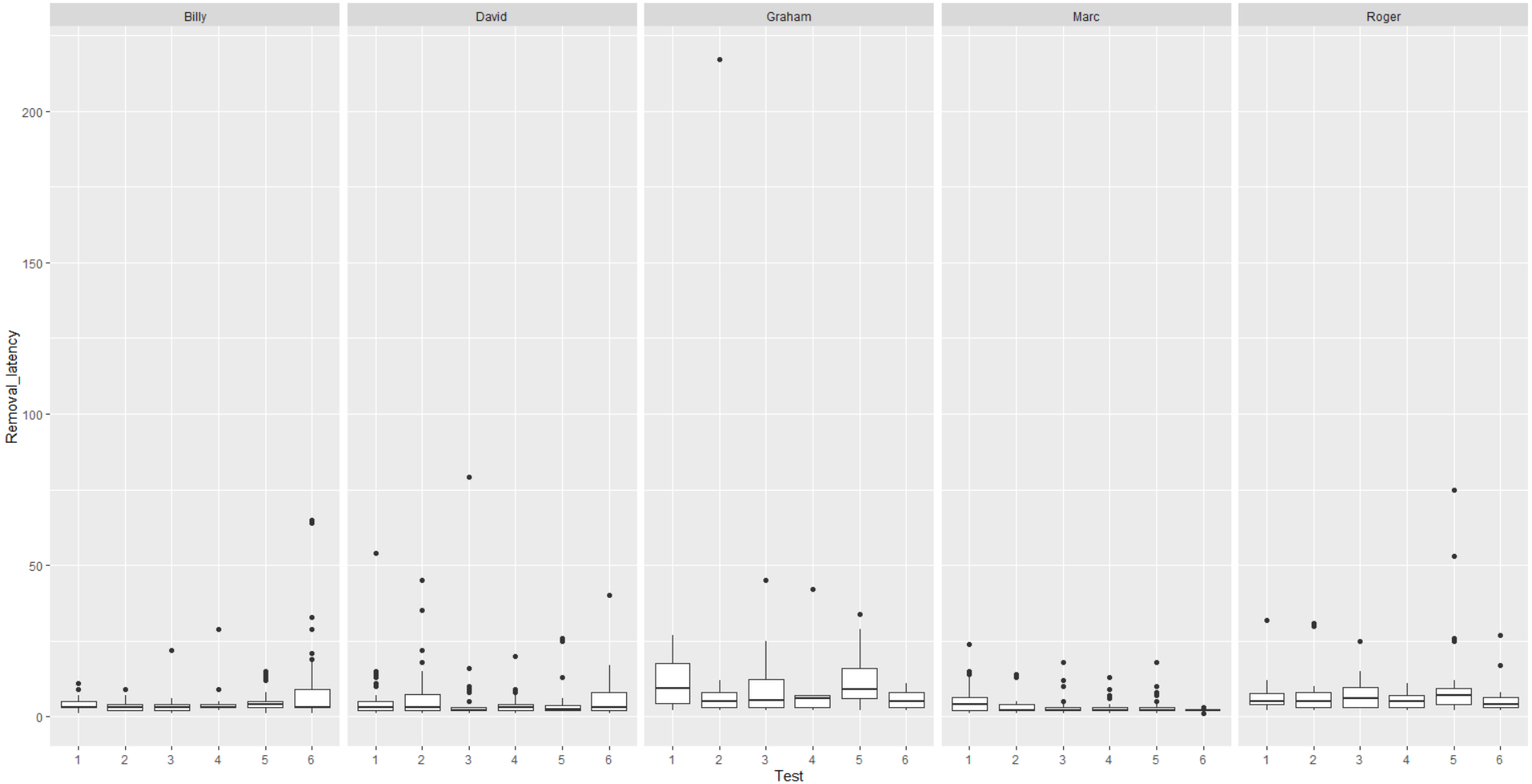


**Supplementary figure 2.** Evolution of the latency to remove objects across tests

|  |  | Estimate ± SE | t-value | p-value |
| --- | --- | --- | --- | --- |
| **Result of the model** | Intercept | 1.81 ± 0.20 | 9.26 | **<0.001 ***** |
|  | Object removed: Stone | 0.03 ± 0.06 | 0.53 | 0.60 |
|  | Model AICc | 4506.4 | | |
|  | Null model AICc | 4504.6 | | |

**Supplementary table 7.** Results of a generalised linear mixed-effect model (Removal latency ~ Test epoch + (1|Subject), Gamma family with log link function)

*Across sessions*

*
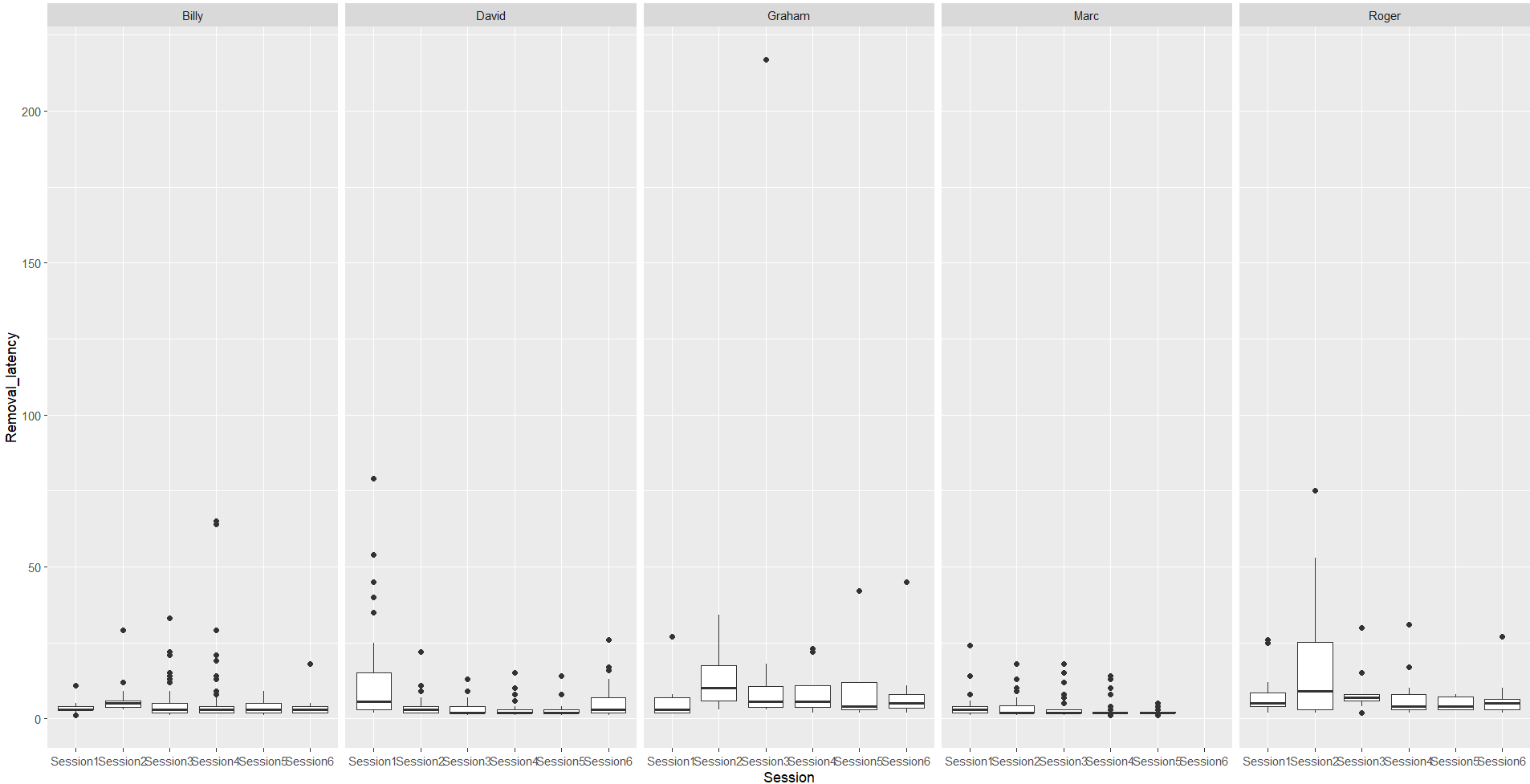
*

**Supplementary figure 3.** Evolution of the latency to remove objects across sessions

|  |  | Estimate ± SE | t-value | p-value |
| --- | --- | --- | --- | --- |
| **Result of the model** | Intercept | 1.97 ± 0.19 | 10.31 | **<0.001 ***** |
|  | Object removed: Stone | -0.36 ± 0.06 | -6.31 | **<0.001 ***** |
|  | Model AICc | 4467.8 | | |
|  | Null model AICc | 4504.6 | | |

**Supplementary table 8.** Results of a generalised linear mixed-effect model (Removal latency ~ Session epoch + (1|Subject), Gamma family with log link function)
